# Urine-based detection of HPV for cervical cancer screening: towards clinical implementation

**DOI:** 10.1128/jcm.00133-26

**Published:** 2026-03-16

**Authors:** Guorong Li, Maryame Lamsisi, Sara Chenafi, Louise Moniod, Abdelhamid Benlghazi, Jaouad Kouach, Céline Chauleur, Thomas Bourlet

**Affiliations:** 1 Department of Urology, North Hospital 26926, CHU Saint-Étienne, France; 2 R&D in Biotechnology Unit, Institut Pasteur du Maroc 290327, Casablanca, Morocco; 3 Laboratory of Virology, North Hospital 26926, CHU Saint-Étienne, France; 4 Department of Obstetrics and Gynecology, North Hospital 26926, CHU Saint-Étienne, France; 5 Department of Obstetrics and Gynecology, Mohammed V Military Teaching Hospital, Rabat, Morocco; Mayo Clinic Minnesota, Rochester, Minnesota, USA

**Keywords:** urine, HPV detection, cervical cancer screening, clinical implementation

## LETTER

Cervical cancer continues to be a major global health issue, particularly in low- and middle-income countries where access to effective screening and treatment remains limited (1). The primary cause of cervical cancer is persistent infection with high-risk human papillomavirus (HPV) types, especially HPV 16 and 18. Early detection of HPV, particularly high-risk strains, is crucial for preventing the progression to cervical cancer. In response to the need for more accessible and efficient screening methods, urine-based HPV testing has emerged as a promising non-invasive alternative to traditional cervical screening approaches, such as Pap smears and HPV DNA testing (2, 3). A recent study by Manjeshwar et al. offers an innovative method for detecting HPV in urine, which could improve access to cervical cancer screening (4).

We and other experts have advocated for the standardization of urine-based HPV testing to facilitate its implementation in clinical laboratories (5). Among the key challenges in advancing this technology, sensitivity remains the most critical factor. Sensitivity can be influenced by various variables, such as the volume and timing of urine collection. HPV infects the epithelial cells of the cervix; viral DNA and infected cells are shed into the urine. The first-void urine—the initial portion of urine expelled during urination (typically 20–30 mL)—is often preferred for testing, as it tends to contain the highest concentration of HPV DNA (6). Therefore, standardizing collection protocols across diverse populations and healthcare settings is essential to ensure reliable results. Any variability in sample quality could impact test accuracy, which, in turn, could undermine the test’s overall effectiveness. In the study by Manjeshwar et al., 100 mL of first-void urine was collected, but the volume may have diluted the HPV DNA, potentially reducing sensitivity (4). Although a substantial volume (40 mL) was analyzed in their study (4), determining the optimal urine volume for maximum sensitivity remains a subject for further investigation.

Another challenge is the standardization of detection protocols. Currently, widely used PCR-based systems, such as Cobas, are employed in clinical laboratories for HPV detection in cervical samples. These PCR systems can also be adapted for use with urine samples (7). However, while urine-based testing can be conducted in low-resource settings, the availability of affordable, reliable diagnostic tests for HPV in urine remains a limitation. Ensuring that diagnostic kits and platforms are accessible and cost-effective in remote or under-resourced regions is crucial for widespread implementation. In the study by Manjeshwar et al., next-generation sequencing (NGS) was employed to optimize the detection of HPV in urine samples (4). NGS is a high-throughput, highly sensitive technique capable of detecting a broad range of HPV types, including rare or hard-to-identify strains. HPV genotyping is directly relevant to clinical applications for the development of cervical cancer screening assays (8). However, despite its sensitivity, NGS remains relatively expensive and requires specialized technical expertise, which may limit its widespread adoption in routine clinical practice. Other diagnostic approaches, such as isothermal amplification techniques, are also being explored for HPV detection in urine, with the potential to provide more affordable and simpler alternatives suitable for implementation in low- or middle-income countries (9, 10).

While current evidence supporting the sensitivity and specificity of urine-based HPV testing is strong (2, 4), several challenges remain, including the need for standardized protocols, reliable infrastructure, and further validation studies. The clinical implementation of urine-based HPV testing is feasible, but its widespread adoption will require careful attention to technical, logistical, and regulatory factors. Ultimately, the success of this new screening method will depend on the development of robust guidelines, increased access to testing, and ensuring that patients are well informed about the benefits and limitations of this promising new technology.
